# Supplementary figures and images for: Inhibition of cytosine 5-hydroxymethylation during progression of cancer precursor lesions in the uterine cervix
Source: PLoS One. 2024 Apr 18;19(4):e0297008. doi: 10.1371/journal.pone.0297008 (PMC11025792; doi:10.1371/journal.pone.0297008)

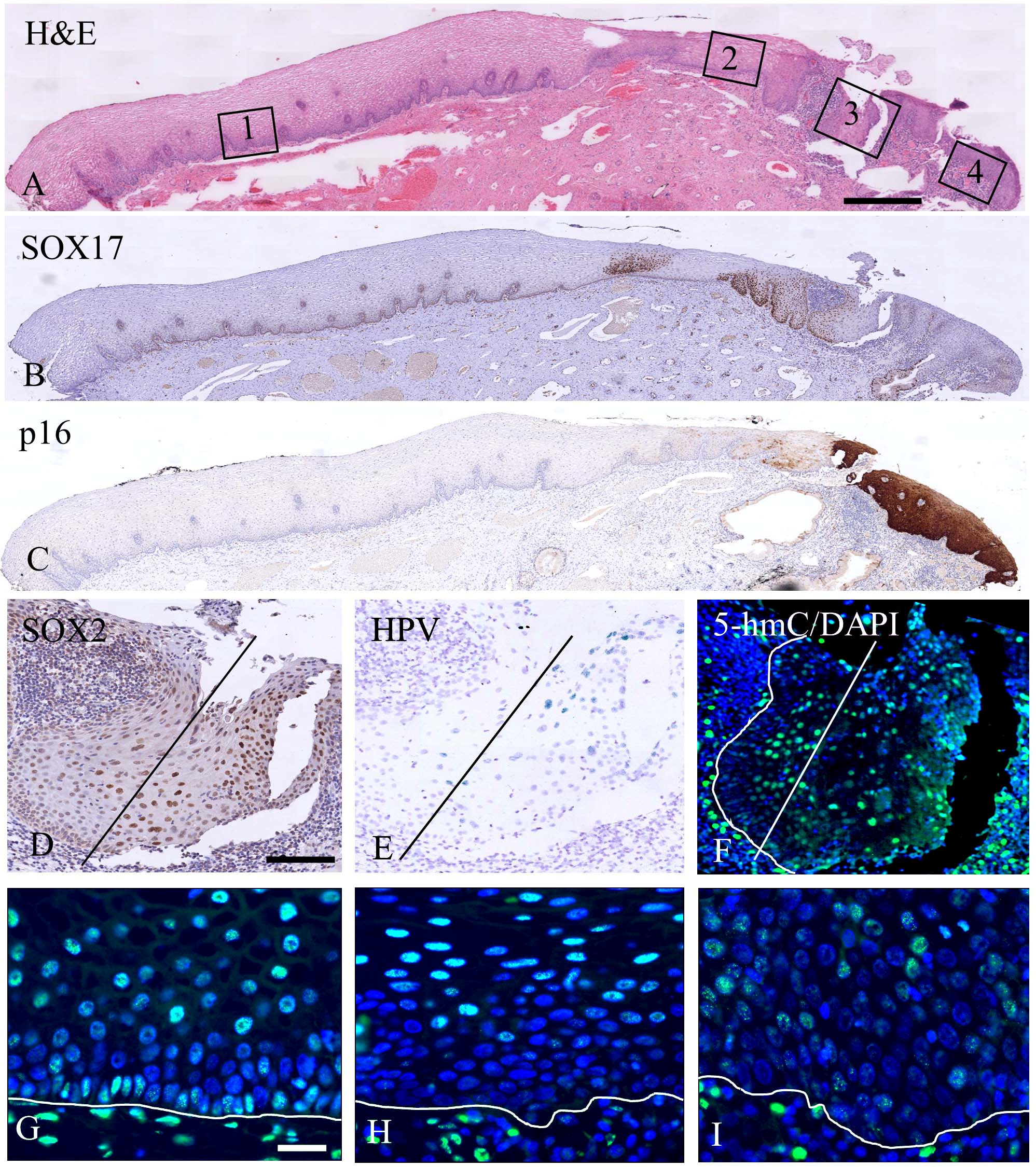

Supplement: S1 Fig — A) H&E-stained stretch of squamous epithelium. B) Immunostaining of immature metaplastic epithelium by SOX17 and C) immunostaining of p16 for HPV-infected CIN3. D-F) Higher magnifications of boxed area 3 indicated in A, showing SOX2 immunostaining (D), HPV detection by chromogenic in situ hybridization (E) and 5-hmC immunofluorescence (F). Straight lines in D-F mark a collision area with the left area being SOX2 negative, HPV negative and 5-hmC weak to negative in the basal/parabasal compartment of the immature metaplastic epithelium. In the right area SOX2 stained positive, HPV is detected and 5-hmC shows an overall weak to negative staining and scattered positive nuclei. The lines in F-I indicate the position of basement membrane. G-I) Higher magnifications of boxed areas 1, 2 and 4, respectively, showing 5-hmC immunostaining. In the area distal (box 1) from the infected TZ (box 3) the basal cells showed a high fluorescence for 5-hmC (compare Fig 1D). In the area in box 4, the dysplastic epithelium showed a weak staining for 5-hmC (compare Fig 6K). Scale bars indicate 500 μm in A (same magnifications in B and C), 100 μm in D (same magnifications in E-F), and 20 μm in G (same magnifications in H and I). (TIF) [file pone.0297008.s001.tif]

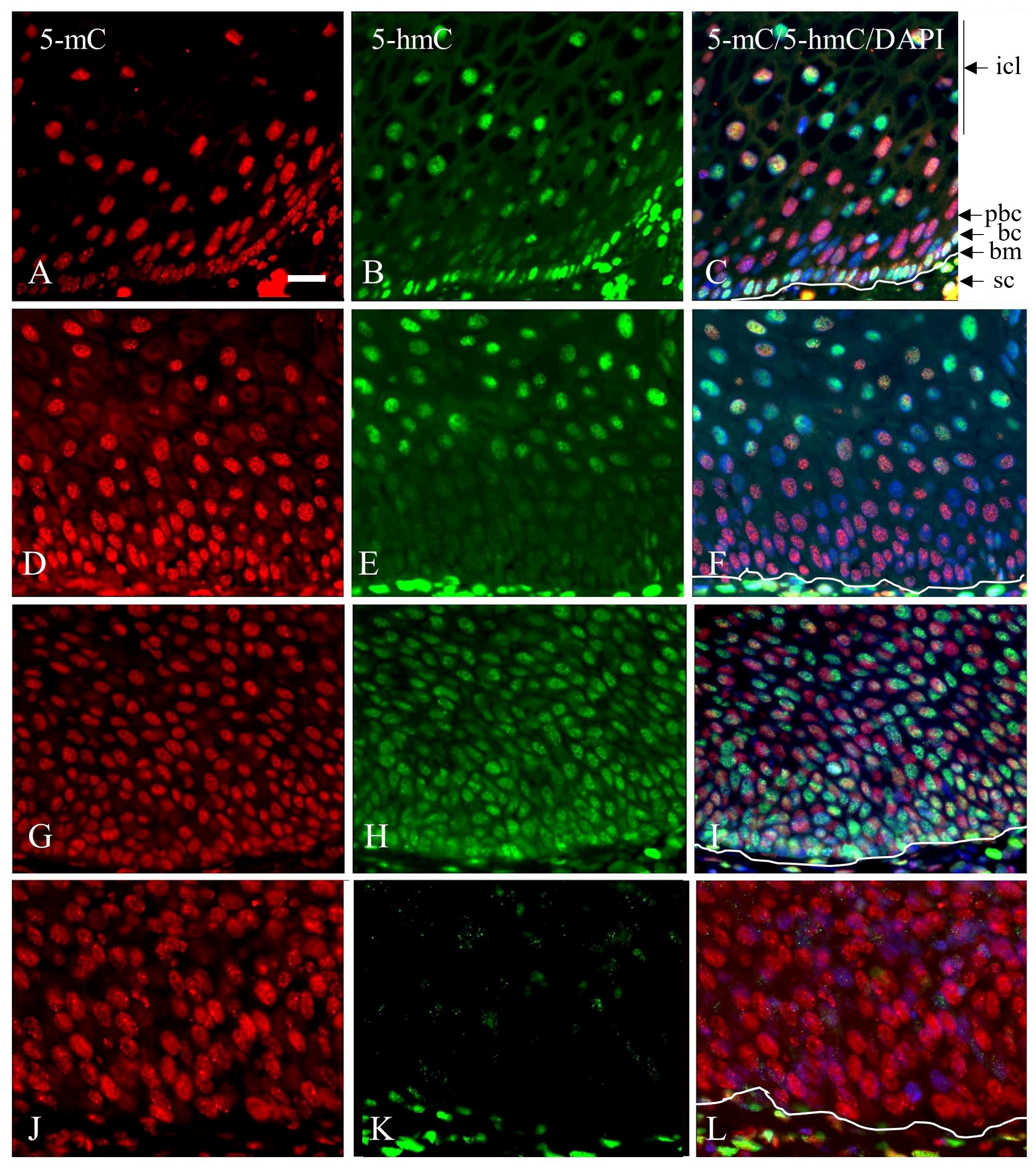

Supplement: S2 Fig — A-C) CIN1 area in CIN1 patient; D-F) CIN1 area in CIN3 patient; G-I) CIN3 area diagnosed in CIN3 patient; J-L) CIN3 area in CIN3 patient. Images were captured using a 40 times oil objective and are higher magnification images of cases shown in Fig 6. A, D, G and J) 5-mC; B, E, H and K) 5-hmC; C, F, I and L) merged images of 5-mC and 5-hmC with DAPI counterstaining. Note the different staining patterns for 5-hmC with a variation in the basal/parabasal layers being strongly positive to weakly positive or even negative. Scale bar in A indicates 20 μm (same magnifications in B-L). The lines in C, F, I and L mark the position of the basement membrane (bm). The stromal cell layer, basal and parabasal cells and intermediate cell layers are indicated as sc, bc, pbc and icl respectively. (TIF) [file pone.0297008.s002.tif]

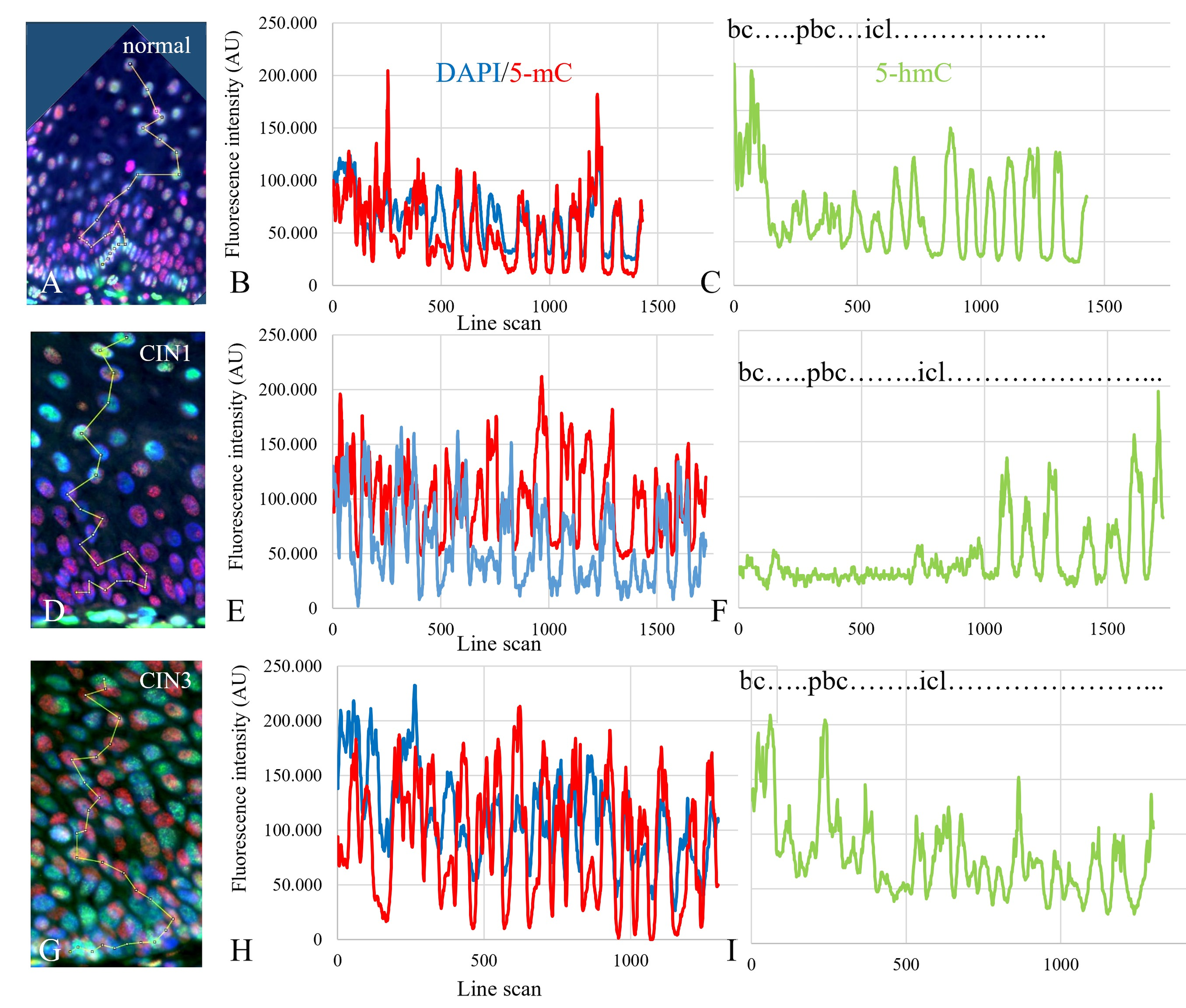

Supplement: S3 Fig — A, D, and G) Merged immunofluorescence images of 5-mC (red), 5-hmC (green) and DAPI (blue) in normal squamous epithelium, CIN1 and CIN3. Lines depicted in A, D and G show the immunofluorescence scan track for the three individual colors from the lower to the higher compartment of the epithelium. B, E and H) Intensity plots for DAPI and 5-mC. C, F and H Intensity plots for 5-hmC. C shows an example of the pattern with a high intensity staining for 5-hmC in the basal cell layer (bc layer) with a reduced intensity in the parabasal cell layer (pbc layer). The intensity for 5-hmC increases in the direction of differentiation of the epithelium to the more upper layer of the epithelium (intermediate cell layer; icl). F) shows an example of the pattern with a low intensity staining for 5-hmC in the lower compartment of the epithelium including the bc, pbc layers and lower compartment of the icl. Compare the difference in fluorescence intensity distribution between 5-mC and 5-hmC. I) shows an example of the pattern with a complex staining for 5-mC and 5-hmC throughout the epithelium, with a strong staining at the bc layer for both 5-mC and 5-hmC, and quantitatively supporting the observed inter- and intra-nuclear differences in immunostaining for 5-mC and 5-hmC. Note that the stromal compartment always shows cells with a high nuclear intensity staining for 5-hmC (see also Figs 1 and 2), which are localized underlying the epithelial basal cell compartment (see S3D Fig). These stromal cells have not been included in this quantitative analysis (see S3E and S3F Fig). (TIF) [file pone.0297008.s003.tif]

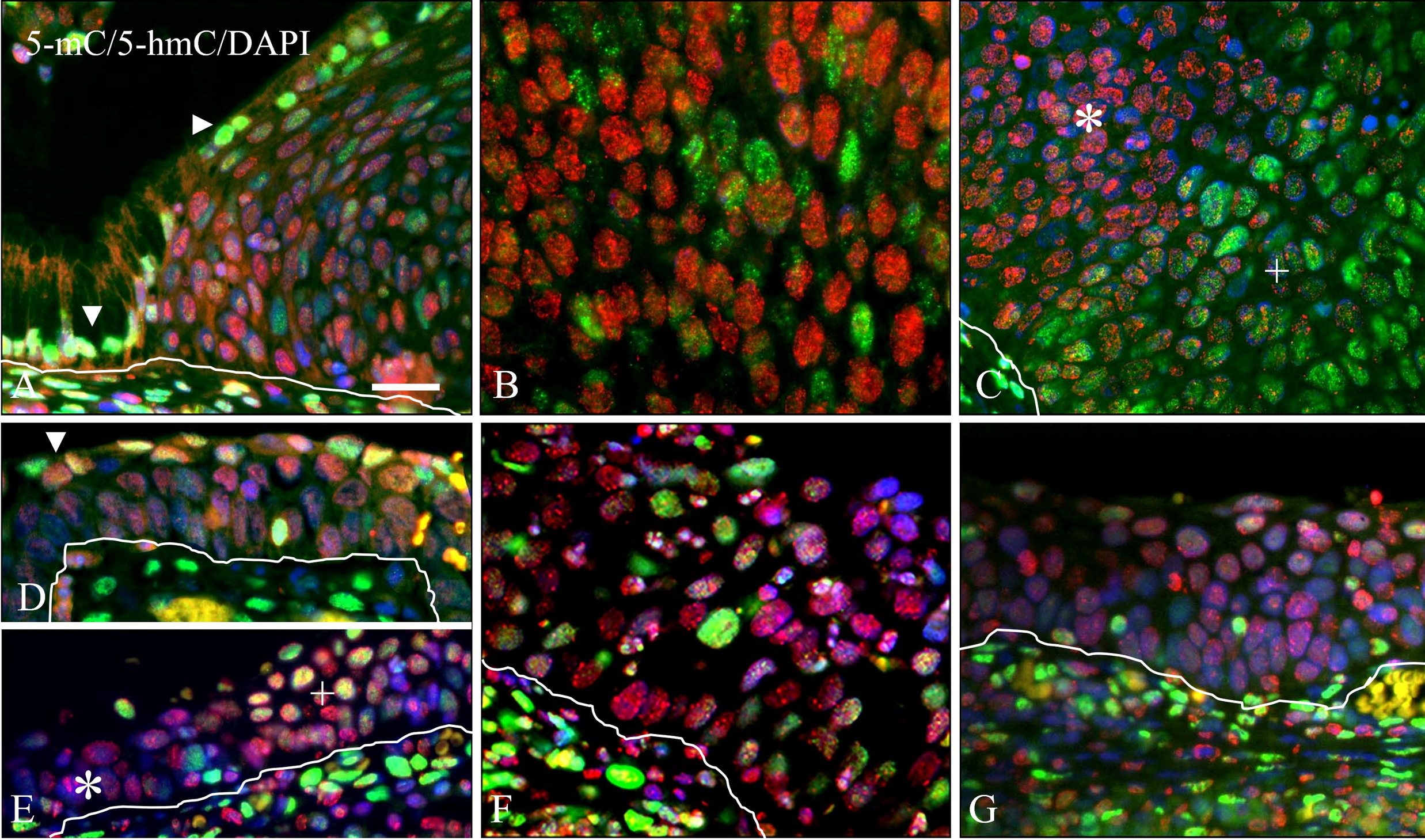

Supplement: S4 Fig — A-G) Merged images of 5-mC in red and 5-hmC in green with DAPI counterstaining in blue. Two patient samples are shown, one in A-C and the other in D-G. Scale bar in A indicates 20 μm (same magnifications in B-G). The lines mark the position of the basement membrane. In A and D arrowheads point to normal columnar epithelium and columnar epithelium on top of the dysplastic squamous epithelium. In C and E +: indicates areas with an increased 5-hmC immunostaining as compared to * indicating areas with a decrease of 5-hmC immunostaining. (TIF) [file pone.0297008.s004.tif]

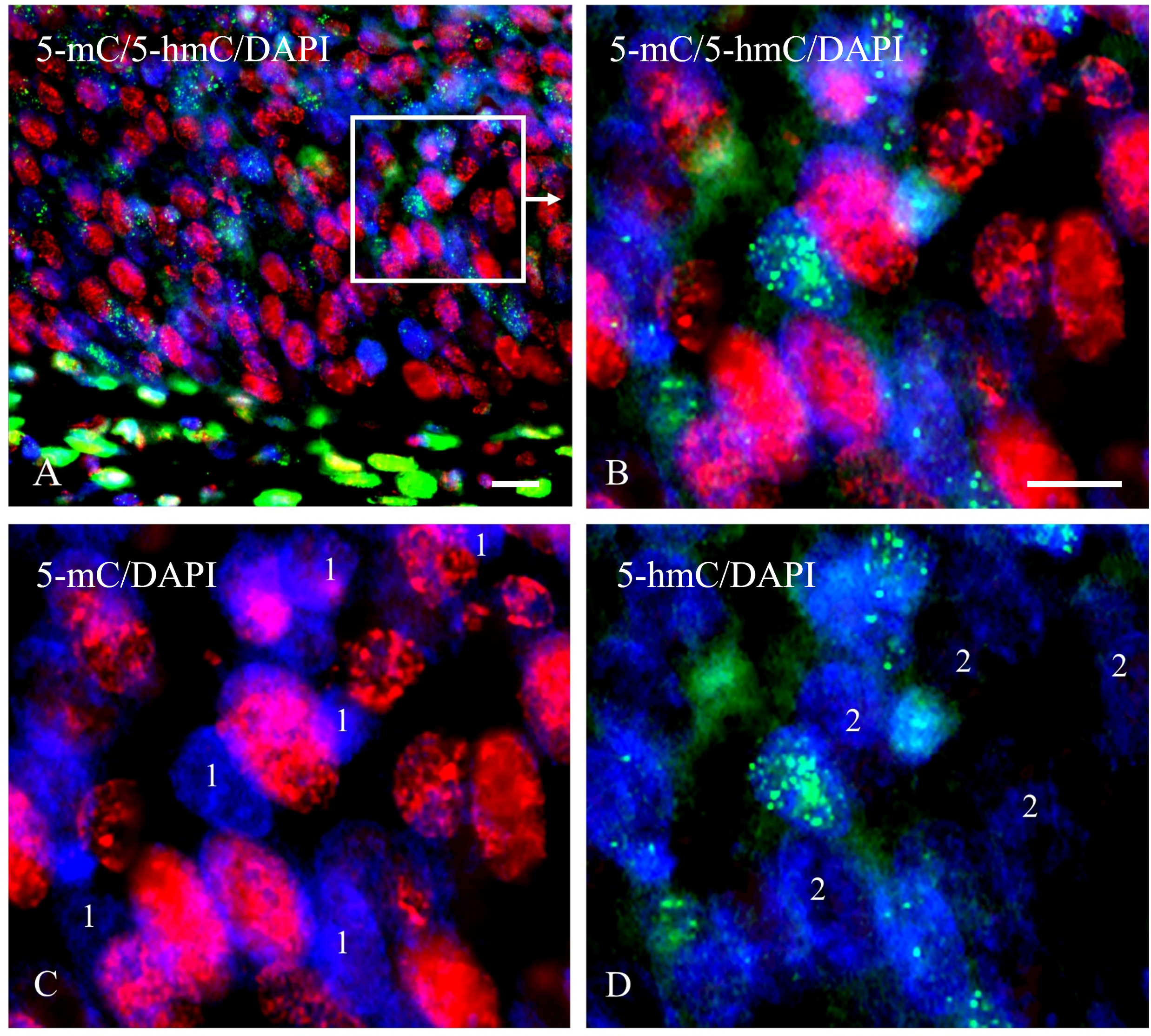

Supplement: S5 Fig — A) Merged image of 5-mC in red and 5-hmC in green with blue DAPI counterstaining; B) Higher magnification of boxed area in A; C) 5-mC with blue DAPI counterstaining. D) 5-hmC with blue DAPI counterstaining. Note the mutually exclusive staining patterns for 5-mC and 5-hmC. Nuclei with dominant 5-hmC staining indicated with 1 and 5-hmC dominant staining indicated with 2. Scale bar in A indicates 10 μm and 20 μm in B (same magnifications in C and D). (TIF) [file pone.0297008.s005.tif]

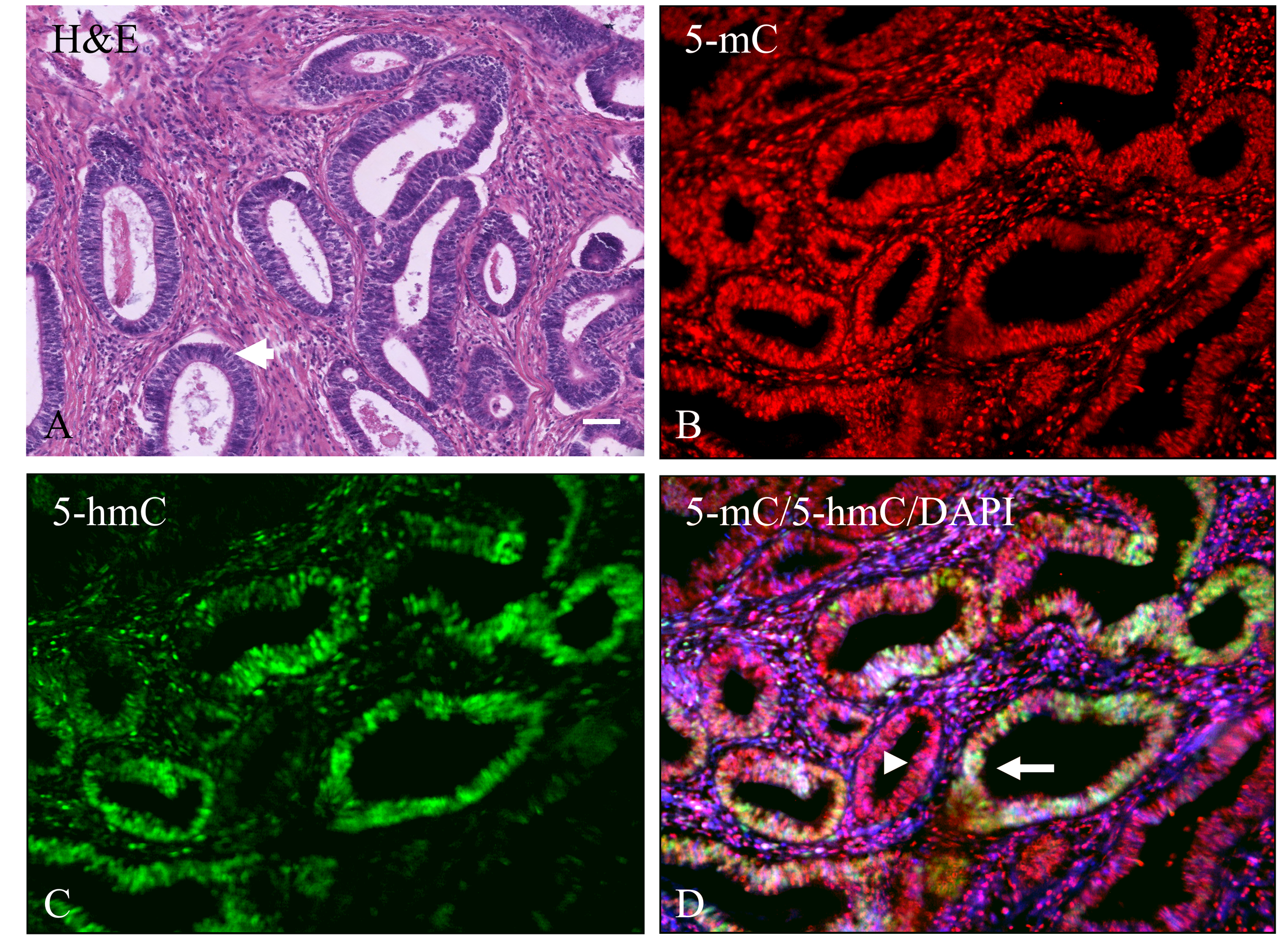

Supplement: S6 Fig — A) H&E-stained dysplastic glands in adenocarcinoma. B) 5-mC; C) 5-hmC; D) merged mages of 5-mC and 5-hmC with DAPI counterstaining. Scale bar in A indicates 100 μm (same magnifications in B-D). (TIF) [file pone.0297008.s006.tif]
