# Supplementary material for: Inhibition of cytosine 5-hydroxymethylation during progression of cancer precursor lesions in the uterine cervix
Source: PLoS One. 2024 Apr 18;19(4):e0297008. doi: 10.1371/journal.pone.0297008 (PMC11025792; doi:10.1371/journal.pone.0297008)
Supplement: S1 Table — A Antibody characteristics and optimized detection methods applied for the single- and double-label immunofluorescence analyses of 5-mC and 5-hmC. B Antibody characteristics and optimized detection methods applied for the bright field immunohistochemical analyses. (PDF) [file pone.0297008.s007.pdf]

S1A Table. Antibody characteristics and optimized detection methods applied for the single- and double-label immunofluorescence analyses of 5-mC and 5-hmC

Antigen	Primary Antibody	Dilution	Secondary antibody
5-mC	mAb Mouse IgG1<br>Clone 33D3<br>Eurogentec, Seraing, Belgium	1:100 PBST/5% NGS,<br>30 min, 37^0C	TexasRed conjugated Goat anti Mouse Ig, SouthernBiotech, Birmingham, UK<br>1010-07<br>1:100 in PBST/5% NGS<br>30 min, 37^0C
5-mC	mAb Mouse IgG1<br>Clone 33D3<br>Eurogentec, Seraing, Belgium	1:100 in PBST/5% NGS,<br>30 min, 37^0C	AlexaFluor 555 conjugated Goat F(ab')2 anti Mouse Ig, SouthernBiotech, Birmingham, UK<br>1012-32<br>1:100 in PBST/5% NGS<br>30 min, 37^0C
5-hmC	mAb Rabbit IgG<br>Clone RM236<br>Ab214728<br>Abcam, Cambridge, UK	1:100 in PBST/5% NGS,<br>30 min, 37^0C	FITC conjugated Goat anti Rabbit IgM and IgG, SouthernBiotech, Birmingham, UK<br>4010-02<br>1:100 in PBST/5% NGS<br>30 min, 37^0C

Abbreviations: mAb, monoclonal antibody; PBST, phosphate buffered saline containing 0.1% Tween-20 (Janssen Chimica, Beerse, Belgium); NGS, normal goat serum. For the double-label immunofluorescence studies a combination of AlexaFluor 555 conjugated Goat F(ab')2 anti Mouse Ig and FITC conjugated Goat anti Rabbit IgM and IgG was applied as secondary antibodies.

S1B Table. Antibody characteristics and optimized detection methods applied for the bright field immunohistochemical analyses

Antigen	Primary Antibody	Dilution	Secondary antibody	Enhancement/ Detection
SOX2	pAb Goat IgG<br>AF2018<br>R&D Systems, Abbingdon,UK	1:100 in PBST/1% BSA,<br>1 hr RT	Biotinylated Horse anti Goat IgG, Vector Laboratories, Burlingame, CA, USA,<br>BA-9500<br>1:200 in PBST/1% BSA;<br>30 min RT	ABC, Vectastain Elite ABC Kit, Vector Laboratories, Burlingham, CA, USA,<br>30 min RT<br>DAB reaction
SOX2	mAb Rabbit IgG<br>clone EPR3131<br>Ab92494<br>Abcam<br>Cambridge UK	1:100 in PBST/5% NGS,<br>45 min,<br>37^0C	Biotinylated Goat anti Rabbit IgG, BA-1000<br>1:200 in PBST/5% NGS<br>30 min, 37^0C	ABC, Vectastain Elite ABC Kit, Vector Laboratories, Burlingham, CA, USA,<br>30 min RT<br>DAB reaction
SOX17	pAb Goat IgG<br>GT15094<br>Neuromics, Edina, MN, USA	1:2500 in PBST/1% BSA,<br>1 hr RT	Biotinylated Horse anti Goat IgG, Vector Laboratories,<br>BA-9500<br>1:200 in PBST/1% BSA;<br>30 min RT	ABC<br>30 min RT<br>DAB reaction
p16	mAb Mouse IgG2a<br>Clone E6H4<br>CINtec, MTM Laboratories AG, Heidelberg, Germany	1:50 in PBST/1% BSA,<br>1 hr RT	Biotinylated Horse anti Mouse IgG, Vector Laboratories,<br>BA-2001<br>1:200 in PBST/1% BSA;<br>30 min RT	ABC<br>30 min RT<br>DAB reaction

Cytokeratin 7	mAb Mouse IgG1<br>Clone OVTL12/30<br>MUB0316P<br>Nordic-MUbio, Susteren, The Netherlands	1:100 in PBST/1% BSA<br>45 min, 37^0C	Biotinylated Horse anti Mouse IgG, BA-2001<br>1:200 in PBST/1% BSA<br>30 min, 37^0C	ABC<br>30 min RT<br>DAB reaction
Cytokeratin 17	mAb Mouse IgG2b<br>Clone E3<br>MUB0325P<br>Nordic-MUbio, Susteren, The Netherlands	1:200 in PBST/1% BSA,<br>1 hr RT	Poly-HRP Goat anti Mouse/Rabbit IgG, Immunologic, Duiven, The Netherlands, Undiluted<br>30 min 37°C	None<br>DAB reaction
Ki-67	mAb Mouse IgG1<br>Clone MIB-1<br>M7240<br>DAKO, Glostrup, Denmark	1:25 in PBST/1% BSA,<br>1 hr RT	Biotinylated Horse anti Mouse IgG, Vector Laboratories,<br>BA-2001<br>1:200 in PBST/1% BSA;<br>30 min RT	ABC<br>30 min RT<br>DAB reaction
5-mC	mAb Mouse IgG1<br>Clone 33D3<br>Eurogentec, Seraing, Belgium	1:500 in PBST/5% NGS,<br>30 min, 37^0C	Biotinylated Horse anti Mouse IgG, Vector Laboratories,<br>BA-2001<br>1:200 in PBST/1% BSA;<br>30 min 37^0C	ABC<br>30 min RT<br>DAB reaction
5-hmC	mAb Rabbit IgG<br>Clone RM236<br>Ab214728<br>Abcam, Cambridge, UK	1:100 in PBST/5% NGS,<br>30 min, 37^0C	Biotinylated Goat anti Rabbit IgG, Vector Laboratories, BA-1000<br>1:200 in PBST/5% NGS<br>30 min, 37^0C	ABC<br>30 min RT<br>DAB reaction

Abbreviations: mAb, monoclonal antibody; pAb, polyclonal antibody; HRP, horseradish peroxidase; RT, room temperature; PBST, phosphate buffered saline containing 0.1% Tween-20 (Janssen Chimica, Beerse, Belgium); BSA, bovine serum albumin; DAB, diaminobenzidine; ABC, Avidin-biotin complex with biotinylated HRP; NGS, normal goat serum.
