# Supplementary material for: Inhibition of cytosine 5-hydroxymethylation during progression of cancer precursor lesions in the uterine cervix
Source: PLoS One. 2024 Apr 18;19(4):e0297008. doi: 10.1371/journal.pone.0297008 (PMC11025792; doi:10.1371/journal.pone.0297008)
Supplement: Attachment — Submitted filename: Figure_integration.pdf [file pone.0297008.s009.pdf]

Inhibition of cytosine 5-hydroxymethylation during progression of cancer precursor lesions in the uterine cervix

1. normal squamous epithelium

- normal squamous ectocervical epithelium
  - Intense staining is frequently seen in the basal cell layer which differs in intensity from the layers above up to the intermediate cell layer(this pattern was seen in 21 out of 73 normal areas analyzed)

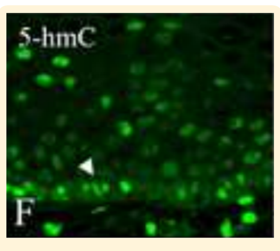

Fig 1F

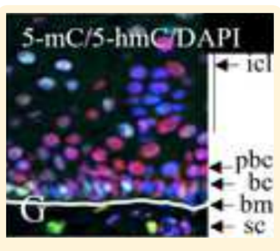

scl: superficial cell layers
icl:intermediate cell layers
pbc:parabasal cells
bc:basal cells
bm:basement membrane
sc:stromal cells

- epithelium in areas more closely located to the squamo-columnar junction (SCJ)
  - more intense 5-hmC staining in the intermediate cell layer as compared to the basal/parabasal layers

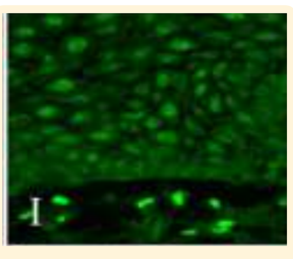

Fig 1I

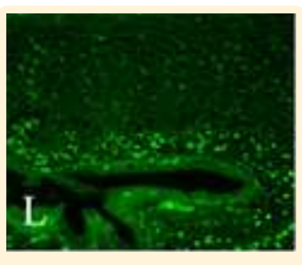

Fig 1L

- in the squamo-columnar junction (SCJ)
  - weak staining is seen in the basal/parabasal layer compared to the superficial layers

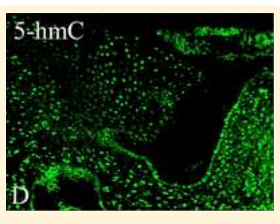

Fig 2D

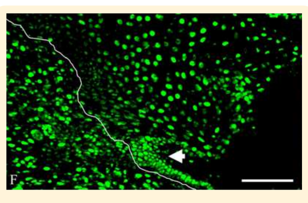

Fig 2F: Higher magnification of 5-hmC immunostaining as shown in Fig 2D

- (immature) metaplastic squamous epithelium
  - Immunostaining for 5-hmC was mainly seen in the intermediate and upper compartment and was weak to negative in the basal/parabasal layers

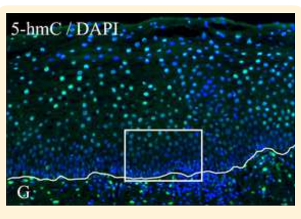

Fig 3G

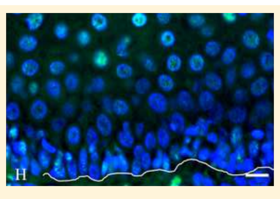

Fig3 H: Higher magnification of the boxed area indicated in Fig3 G

2. normal columnar epithelium and reserve cells

- normal endocervical columnar epithelium
  - The epithelial nuclei showed a significant heterogeneity in staining along the trajectory of the columnar epithelium

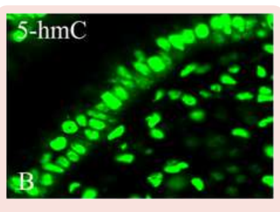

Fig 4B

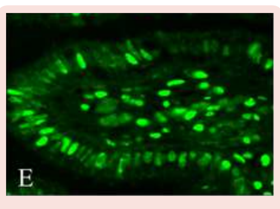

Fig 4E

- normal glandular epithelium and underlying reserve cells
  - These reserve cells are weak or even negative for 5-hmC

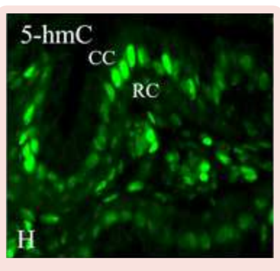

Fig 5H

CC:columnar
RC: reserve cells

3. SIL

- CIN1 and CIN2
  - We want to stress that CIN1 lesions exhibited similar patterns for 5-mC and 5-hmC as recorded for the normal squamous epithelium (Figs 6A-D; see also Supplemental informationvS2 Fig), with intense (3 out of 8 areas analyzed) to weak (5 out of 8 analyzed) staining in thevbasal compartment and positive immunostaining in the intermediate and superficial layers. Ontop of these distributions both 5-mC and 5-hmC positive nuclei were intermingled with weaklyvstained and negative nucleivrecognized in 5 out of 8 CIN1 areas analyzed. These differencesvwere most pronounced for 5-hmC as compared to 5-mC.

    The distribution pattern as shown for CIN1 in Figs 6A-D, exhibiting the low intensity for 5-hmC in the basal compartment, was frequently observed in patients diagnosed with a higher CIN grade,as illustrated in Figs 6E-H, showing weak staining in the lower compartment, but a higher reactivity in the upper layers. This pattern was recognized in 30 out of 84 areas graded higher than CIN1.

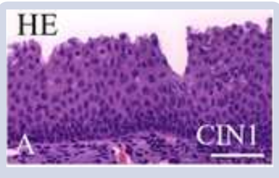

Fig. 6A CIN1

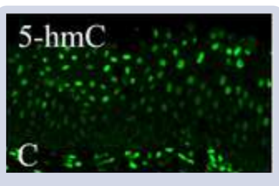

Fig. 6C CIN1

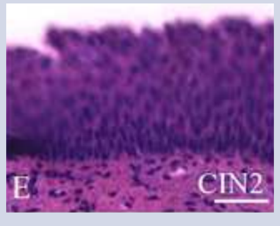

Fig. 6E CIN2

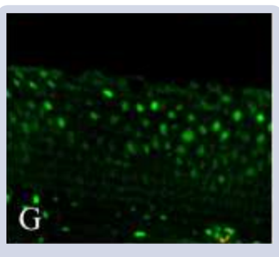

Fig. 6G CIN2

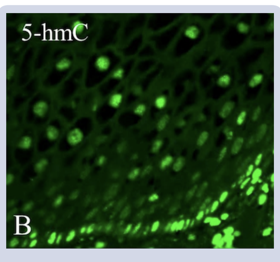

S2 Fig:CIN1 area in CIN1 patient

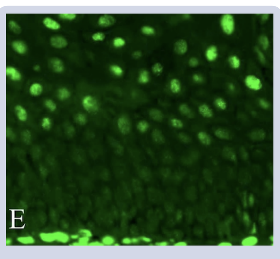

S2 Fig:CIN1 area in CIN3 patient

- CIN3
  - Most CIN2 and CIN3 areas showed for both 5-mC and 5-hmC immunostaining throughout the entire epithelium, next to the low intensity staining for 5-hmC in the lower compartment of the epithelium （Figs 6 I and L; 34 out of 84 areas）

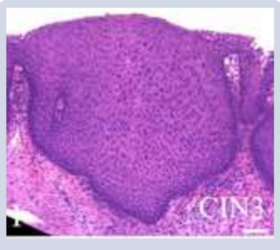

Fig. 6I CIN3

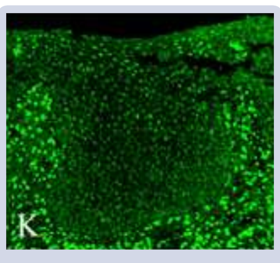

Fig. 6K CIN3

  - about 20% of the patients diagnosed with CIN3 (5 out of 25 patients), a (very) weak staining for 5-hmC was observed throughout the entire epithelium

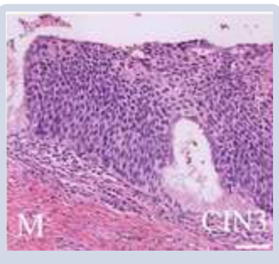

Fig. 6M CIN3

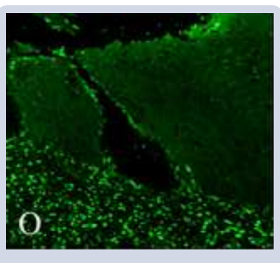

Fig. 6O CIN3

- As shown in Supplemental information S3 Fig these quantitative analyses support the semiquantitative observations that largely three different 5-hmC distribution patterns can be distinguished in CIN lesions
  - 1) a pattern with a high intensity staining for 5-hmC in the basal cell layer, a reduced intensity in the parabasal cell layer and increasing intensity in the direction of differentiation of the epithelium to the more upper layers of the epithelium seen in CIN1;

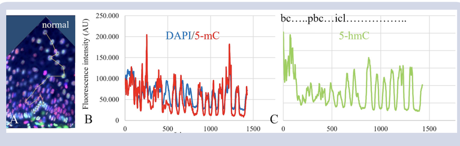
S3 Fig Normal

  - 2) a pattern with a low intensity staining for 5-hmC in the lower compartment of the epithelium including the bc, pbc layers and lower compartment of the icl seen in CIN1-3

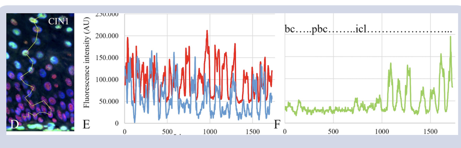
S3 Fig CIN 1

  - 3) a complex staining pattern for 5 mC and 5-hmC throughout the epithelium, with a strong staining at the bc layer for both 5mC and 5-hmC seen in CIN2-3

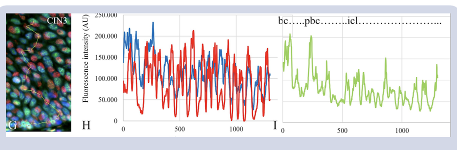
S3 Fig CIN 2

- studying CIN3 cases in more detail
  - combinations of the patterns described above were found next to each other(20 out of 55 areas; see also Supplemental information S4 Fig for examples)
    - CIN3 lesions with overlying columnar epithelium, showing extensive heterogeneity for methylation and hydroxymethylation

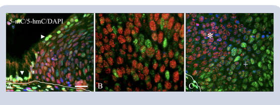
S4 Fig CIN 3 patient one sample

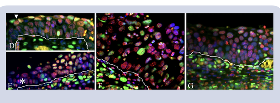
S4 Fig CIN 3 patient two sample

CIN lesions showed a heterogeneous staining pattern for 5-hmC

4. premalignant endocervical glandular lesions

- compared to the normal glandular structures, a drastic decrease in 5-hmC immunostaining was observed in nearly all regions with AIS. Small areas of normal glandular epithelium inside the AIS did, however,show the typical, strong 5-hmC immunoreactivity

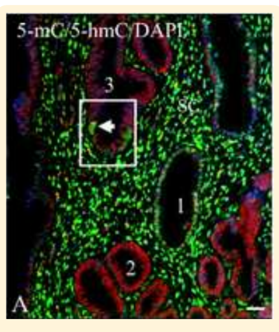

Fig 7A

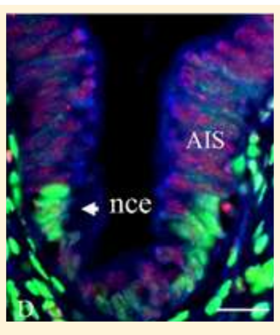

Fig 7D: higher magnifications of Fig 7A

- different patients some variation is seen for 5-hmC staining intensity, but in all 12 AIS cases a drastic decrease of hydroxymethylation was obvious

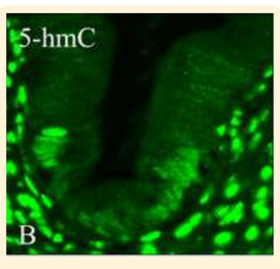

Fig 8B

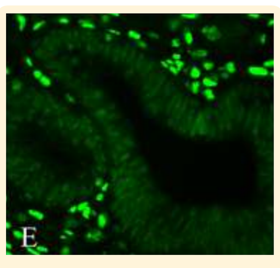

Fig 8E

5. cervical adenocarcinomas

- 12 out of 14 patients showed a greatly reduced immunostaining for 5-hmC, indicating a strong reduction of hydroxymethylation in cervical adenocarcinomas

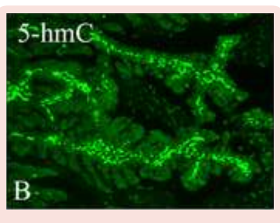

Fig 9B

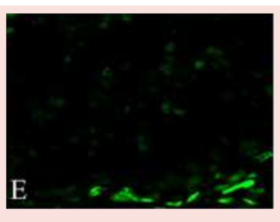

Fig 9E: higher magnification of Fig 9B

- In a minor fraction of the adenocarcinoma cases (2 out of 14), however, an intense staining for 5-hmC was seen intense staining for 5-hmC was seen

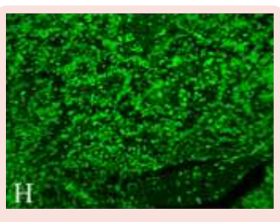

Fig 9H

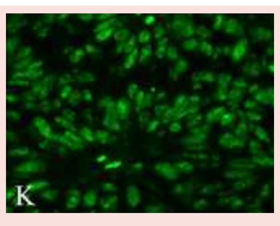

Fig 9K: higher magnification of Fig 9H

Presented with xmind
