# Supplementary material for: Inhibition of cytosine 5-hydroxymethylation during progression of cancer precursor lesions in the uterine cervix
Source: PLoS One. 2024 Apr 18;19(4):e0297008. doi: 10.1371/journal.pone.0297008 (PMC11025792; doi:10.1371/journal.pone.0297008)
Supplement: Attachment — Submitted filename: Responses to reviewer.docx [file pone.0297008.s008.docx]

Rebuttal to the Reviewer’s Comments

First of all, we would like to thank the reviewer for the meticulous assessment of our data and our interpretation of the results. We have therefore made every effort to amend the parts of the manuscript that were commented on. Several new figures now replace the former ones, some of which are included in the Supplemental information paragraph. Also, the text has been adapted and extended to respond to the questions and suggestions of this reviewer.

We can reply to the reviewer’s comments as follows:

**Question 1A**: In Figure 1, in the normal tissue section, whether in the 5-mC staining group or the 5-hmC staining group, we can observe two types of cells: a group of cells with strong staining and a group of cells with weak staining. In Figure 6 and 7, in the abnormal tissue section (CIN), whether in the 5-mC staining group or the 5-hmC staining group, we can also observe groups of cells with strong and weak staining. Therefore, we are curious about the relationship between "staining intensity" and normal or abnormal cells.

**Answer**: We have now performed quantitative analyses of the immunostaining in nuclei from normal and neoplastic cervical epithelium to compare the staining intensities of normal and abnormal cells. These data have been included in the Supplemental information paragraph as S3 Fig and the results have been described in the legend to the figure and summarized in the paragraph dealing with the squamous intraepithelial lesions. It should be realized that comparison of staining intensities amongst different tissues (normal versus neoplastic) are difficult because of the different fixation conditions of the tissues, the accessibility of the antigens, etc. That is why these quantitative analyses can only be used to compare the staining intensities between cells in the same tissue region. The quantitative data can, however, be used to support the semiquantitative observations that largely three different 5-hmC distribution patterns can be distinguished in normal and neoplastic cervical epithelium.

**Question 1B**: Additionally, the authors introduce the concept of "staining pattern". They descripted that "Intense staining is observed in the basal/parabasal layers, which differs in intensity from the upper layers in the part of the normal (?) epithelium shown in box 1 (Fig 1D). The epithelium represented in box 2 and box 3 showed more intense 5-hmC staining in the intermediate and superficial cell layers compared to the basal/parabasal layers (Figs 1G and J)" (Page 10). We think that the description of "staining pattern" carries a strong subjectivity from the authors themselves. Can the "staining pattern" be further divided into different types? Is the "staining pattern" consistent across all samples (60 samples)? To what extent can the "staining pattern" replace the results of “HE staining”? We understand that scientific research requires quantitative descriptions. Results with excessive subjectivity make it difficult to accurately classify different stages of diseases. The authors need to delve into these important questions in order to answer the relationship between the "staining intensity" and "staining pattern" of 5-mC and 5-hmC with the cell types of the disease and disease progression.

**Answer**: We have taken several steps to refute the image of subjectivity when it comes to the “staining pattern” terminology.

- Firstly, at the beginning of the Results section we have now included a short paragraph to explain the difference and relationship between the terms "staining intensity" and "staining pattern" of 5-mC and 5-hmC in normal and preneoplastic tissues.
- Secondly, we refer to our earlier study (reference 25) that describes the staining and distribution patterns of 5-mC and 5-hmC in normal cervical squamous epithelium. This is a study in which we tested several retrieval protocols for an optimal detection of 5-mC and 5-hmC in paraffin sections and performed already quantitative analyses of the staining intensities of cells in the different layers of the normal cervical epithelium.
- Thirdly, in the Results paragraph dealing with the squamous intraepithelial lesions the 3 major types of 5-hmC staining patterns (now designated as distribution patterns) are specified.
- Fourthly, these distribution patterns are not consistent across all 60 patient samples and therefore we have included the number of tissue areas that show the individual patterns. This also replies to the reviewer’s comment urging for a quantitative description to answer the relationship between the staining pattern of 5-mC and 5-hmC with the cell types of the disease and disease progression.

The H&E staining cannot be replaced by the 5-mC or 5-hmC staining pattern. In our study the H&E staining is used as the golden standard for the diagnosis of the CIN lesions in combination with more conventional immunomarkers such as p16, Ki-67, Cytokeratins, SOX2 and SOX17.

**Question 2**: In the figure legend of Fig 6, it is mentioned that the four groups of images correspond to "CIN1 area", "CIN1 area," "CIN3 area", and "CIN3 area". However, in page 15 the authors refer to "CIN2" and "CIN2/3" when describing Figure 6. The figure legend of Fig 6 does not correspond with the text of the manuscript. Further clarification is required to resolve this discrepancy.

**Answer**: We have now included a new Figure 6 into the body of the text and moved the former Figure 6 to the Supplemental information paragraph as S2 Fig. The new Figure 6 shows H&E and immunofluorescence staining images for 5-mC and 5-hmC in a CIN1, a CIN2 and two different patterns for CIN3. Also the body of the text and the legend to figure 6 has been adapted to clarify the apparent discrepance that existed in the former version of the manuscript.

**Question 3**: Page 15. The text mentions "Using H&E staining of the tissue sections of the different patient samples as a guide to recognize specific areas with". The authors should provide H&E staining pathology images and describe the percentage of atypical cells.

**Answer**: H&E staining images are now incorporated into the new Figure 6 for a CIN1, a CIN2 and two different patterns for CIN3. The number of atypical cells, as well as the dysplastic appearance increases with the CIN grade and was used as a diagnostic criterium by the pathologist.

**Question 4**: Page 15. In the text, Figure 6A-C is described as "In the CIN1 lesions, we observed similar patterns for 5-mC and 5-hmC as recorded in the normal squamous epithelium (Fig 6A-C), with intense to weak staining in the basal/parabasal compartment and lesser staining in the intermediate and superficial layers", in which "basal/parabasal compartment, intermediate and superficial layers" correspond to basal cells (bc), parabasal cells (pbc), intermediate cell layers (icl) in figure legend, respectively.
However, the author mentioned "lesser staining in the superficial layers" in the text, while the figure legend states "The superficial cell layer is not shown in the fluorescence images." Please provide additional images that include the "superficial cell layer." This will help ensure consistency between the text and the figure legend and provide a more complete representation of the study's findings.

**Answer**: We have now included a new Figure 6 that contains lower magnifications of immunofluorescence staining patterns for 5-mC and 5-hmC for the different grades of CIN. These images now also include the superficial cell layers. The former Figure 6 with the higher magnification images (and therefore missing the superficial cell layers) is now S2 Fig in the Supplemental information paragraph.

Also, in Figure 1 we have now included lower magnifications of immunofluorescence staining patterns for 5-mC and 5-hmC for the normal cervical epithelium.

**Question 5**: Page 15. Please clarify how to obtain the result that "Most CIN3 areas showed this pattern with weak staining in the lower compartment of the epithelium" of Figs 6 G and H. And the authors should display the immunostaining results for all 5 patients diagnosed with CIN3. Please label the images clearly to indicate their source from each of the five patients. This will help readers understand and assess the consistency of the observed staining patterns across the CIN3 cases.

**Answer**: We believe that there exists some confusion about the number of patients diagnosed with CIN3. As can be seen from Table 1, of the 60 patients included in our study a total of 25 patients was diagnosed with CIN3. In the different neoplastic regions within the tissue sections different 5-hmc immunostaining patterns were observed. The observed numbers of these different patterns in relation to the total number of regions examined have now been included throughout the text. Only 5 of the 25 CIN3 lesions showed the pattern where all layers of the epithelium were negative for 5-hmC pattern. We see no use in depicting specifically these 5 cases with similar staining patterns in a separate image. In Figure 6 and in supplemental figure S2 Fig we have depicted the major 5-mC and 5-hmC staining patterns observed in the different CIN lesions, including an example of these negative cases.

Also, the text in the paragraph dealing with the squamous intraepithelial lesions has been adapted and extended, the quantitative data have been included and the different patterns have been more clearly described.

**Question 6**: Page 15. The author mentioned, "while most nuclei were positive for 5-mC (Fig 6J)", but it is not clear from Figure 6J that the nuclei of the cells are positive. The authors should provide clearer and more representative images or data that demonstrate the positive staining of nuclei for 5-mC. This will help ensure that the findings are accurately represented and can be readily interpreted by readers.

**Answer**: We have now included a new figure 6 with representative images that demonstrate the positive staining of nuclei for 5-mC and its relation to the 5-hmC and DAPI staining patterns. The low magnification images provide a good overview of the total tissue area and should now be easier to interpret by the readers.

The former Figure 6 is now supplementary figure S2 Fig. To show the overlap with the 5-mC staining we have now also slightly increased the intensity of the DAPI image in what was the former Figure 6L and is now supplementary figure S2L Fig.

**Question 7**: Page 26. In the "Clinical utility" section of the Discussion, the authors modified the text as "When interpreting the result of such an analysis one has to realize, however, that the demethylation process not only involves a single step of hydroxymethylation but is a complex cascade that involves multiple stages, including aldehyde methylation and carboxymethylation, which could have a similar effect on this demethylation process [2]".
As mentioned in the reference you cited, it might be more accurate to state that "5-hydroxymethylcytosine (5hmC), 5-formylcytosine (5fC), and 5-carboxylcytosine (5caC)" compared to "cytosine hydroxymethylation", "aldehyde methylation" and "carboxymethylation".

**Answer**: We have now amended the text in the Clinical utility paragraph as follows:

“When interpreting the result of such an analysis one has to realize, however, that in addition to 5-hmC, the Tet proteins can generate 5-formylcytosine (5-fC) and 5-carboxylcytosine (5-caC) from 5-mC. A potential alternative 5-mC demethylation mechanism has therefore been suggested by Ito et al [2], in which the Tet proteins oxidize 5-mC not only to 5-hmC, but also to its aldehyde form 5-fC and the carboxylic acid form 5-caC.“
