# Supplementary material for: Inhibition of cytosine 5-hydroxymethylation during progression of cancer precursor lesions in the uterine cervix
Source: PLoS One. 2024 Apr 18;19(4):e0297008. doi: 10.1371/journal.pone.0297008 (PMC11025792; doi:10.1371/journal.pone.0297008)
Supplement: Attachment — Submitted filename: Responses to reviewer Revision 21_12_2023.docx [file pone.0297008.s010.docx]

Rebuttal to the Reviewer’s Comments

We would like to thank the reviewer for the meticulous assessment of our revision. The pdf entitled: Figure_integration.pdf provides an excellent overview of the 5-hmC staining patterns in relation to the type of (premalignant) epithelia. We would appreciate that the peer review history would be included with our manuscript, including the Figure_integration.pdf from the reviewer.

We can reply to the editor’s and reviewer’s comments as follows:

**Question**: Please review your reference list to ensure that it is complete and correct.

**Answer**: We have reviewed the reference list to ensure that it is complete and correct.

**Question** **Reviewer #1**: Jobran et al.'s responses addressed some of the questions, but there are still certain questions that require further clarification.

¬ In the "Clinical utility" section, "As a consequence... the detection of global hydroxymethylation by means of immunohistochemical staining of 5-hmC may potentially become an important diagnostic tool in the investigation of glandular lesions of the cervix ", The authors concluded that immunohistochemical staining of 5-hmC can be used as an important diagnostic tool for cervical glandular lesions……. Therefore, it is recommended that authors need to add explanations to this issue or revise their conclusion to prevent misleading readers. Or add reasonable explanations in the discussion section to explain the shortcomings of this research work, so as to facilitate readers to understand the innovation points and potential application directions of this research work.

**Answer**: We have adapted the text in the “Clinical utility” section and revised our conclusion as follows.

As a consequence, not only the methylation status of specific genes can be used as an indicator for the degree of malignant transformation of the cervical squamous and glandular lesions [15-17], but also the detection of global hydroxymethylation by means of immunohistochemical staining of 5-hmC. In case of glandular lesions an inhibition of global demethylation was frequently recognized in AIS, the pre-stage of adenocarcinoma, and may become of clinical utility in combination the conventional H&E staining. Such a correlation between the loss of 5-hmC levels and the degree of malignant progression was less evident for the squamous premalignant lesions. For that reason, the potential clinical utility of 5-mC and 5-hmC immunostaining is limited for these squamous lesions. In diagnostically challenging clinical CIN samples, however, the 5-hmC staining patterns, showing either positivity throughout the full thickness of the epithelium or a negative epithelium, are indications for a high-grade lesion in addition to the morphological criteria.

**Question Reviewer #1**: In addition, in "S3 fig R2, D-E-F", the bottom green signal is not measured, so there is no signal peak on the left in figure F. Please confirm whether the author missed this important signal?

**Answer**: We included a note in the legend of Figure S3: Note that the stromal compartment always shows cells with a high nuclear intensity staining for 5-hmC (see also **Figs 1** and **2**), which are localized underlying the epithelial basal cell compartment (see **S3 Fig D**). These stromal cells have not been included in this quantitative analysis (see **S3 Figs E** and **F**).

For the reader we also have added this note to the legends of **Figs 1** and **2**: Note also the intense 5-hmC immunostaining in the stromal cell compartment.
